# Sex-specific association between maternal childhood adversities and offspring’s weight gain in a Brazilian cohort

**DOI:** 10.1038/s41598-025-87078-5

**Published:** 2025-01-23

**Authors:** Vinicius Oliveira Santana, Aline Camargo Ramos, Hugo Cogo-Moreira, Célia Maria Araújo, Barbara Shibuya Alves, Lucas Ribeiro, Aline Lodi, Ana Carolina Coelho Milani, Ivaldo Silva, Cristiane S. Duarte, Jonathan Posner, Andrea Parolin Jackowski

**Affiliations:** 1 Laboratory of Integrative Neuroscience (LiNC), Universidade Federal de São Paulo, São Paulo, Brazil; 2 Department of Psychiatry, Universidade Federal de São Paulo, Rua Pedro de Toledo 669, 3o. andar., São Paulo, SP Brazil; 3 Department of Education, Information and Communications Technology (ICT) and Learning, Østfold University College, Halden, Norway; 4 Department of Gynaecology, Universidade Federal de São Paulo, São Paulo, Brazil; 5 New York State Psychiatric Institute, Columbia University Irving Medical Center, New York, USA; 6 Duke University Medical Center, Durham, NC USA

**Keywords:** Sex differences, Adverse childhood experiences, Weight gain, Obesity, Neurodevelopment, Outcomes research, Paediatric research

## Abstract

Maternal adverse childhood experiences (ACEs) are linked to negative health and developmental outcomes in offspring. However, whether maternal ACEs influence infant weight gain in the first months of life, and if this effect differs by infant sex, remains unclear. This study included 352 full-term newborns from low-risk pregnancies and their mothers in low-income settings in Brazil. Anthropometric data (weight, length, head circumference) and other information (feeding type, offspring sex, family income) were collected at delivery (W0), discharge (W1), and up to 8 weeks postpartum (W2). ACEs were assessed using the CDC-Kaiser Questionnaire, and weight gain was calculated as the difference between W2 and W1, divided by the number of days between measurements. The association between maternal ACEs and offspring weight gain was positive only in male offspring (unstandardized coefficient (male) = 1.82, SE = 0.438, *p* < 0.001); for each 1-point increase in the ACEs score (e.g., from 0 to 1), weight gain increased by 1.8 g/day. These findings indicate that maternal ACEs are associated with increased weight gain in male infants during the first two months of life, potentially increasing the risk of future obesity. Further research is required to investigate the underlying biological mechanisms and their neurodevelopmental implications.

## Introduction

Adverse childhood experiences (ACEs) encompass potentially traumatic situations, including physical, psychological, and sexual abuse; neglect; parental mental illness; scarcity of resources; and family violence^1–4^. Adversities experienced during childhood can impact personal development^5,6^, further extending beyond directly-exposed individuals, as the impact on an exposed parent can be passed onto the offspring^7,8^. Studies of White populations have indicated an impact of maternal ACEs on weight gain at 5 and 12 months of age^9^, obesity risk at 2 and 5 years of age^10^, and inflammatory markers at 9 years^11^. Another study evaluating growth trajectories in Brazilian children revealed an increase in the prevalence of overweight and obesity from 10.9 to 11.8% in boys < 5 years and from 9.6 to 10.5% in girls < 5 years; and from 26.8 to 30.0% in boys > 5 years and from 23.9 to 26.6% in girls > 5 years^12^. This study further identified diet as an important contributing factor, warning of the increase in consumption of ultra-processed foods in recent years, particularly in low- and middle-income countries due to their low cost^12,13^. Excessive weight gain during early childhood is associated with a higher risk of obesity and chronic diseases in adulthood^13–15^. Obesity is a condition resulting from a disrupted energy balance, which is regulated by neural circuits that begin to develop during pregnancy, and mature in the early postnatal periods, when the brain is highly plastic and sensitive to the environment^16^. Studies have shown that obesity from the earliest stages can influence neurodevelopment, increasing the risk of neuropsychiatric disorders throughout life^17^.

Rapid infant weight gain is defined as weight gain above expected values in a short time, and is strongly related to obesity in adulthood^13–15^. Evidence has suggested that rapid weight gain may be associated with early formula feeding, either exclusively or combined with breastfeeding^15^. One literature review showed that weight gain in the first few days of life differed by sex, with boys gaining approximately 3 g/day more than girls according to the growth z-score used by the World Health Organization^18,19^.

One study performing prenatal follow-up of pregnant women in the UK, revealed that the risk of obesity can vary between boys and girls, even after adjusting for factors such as low dairy consumption, cotinine levels in the blood of non-smokers, low richness of facilities, and green spaces during pregnancy^20^. This study further showed that an environment combining these four exposures specifically protects girls against obesity and neurodevelopmental delays, indicating that combining different exposures in multi-exposure profiles, using causal inference, could be useful for identifying at-risk populations^20^.

Maternal stress can interfere with pre and postnatal development^21,22^. Two prior studies evaluating the impact of maternal trauma on offspring body size in the first year of life in a cohort of 100 mother-infant dyads from Poland demonstrated an increase of 10% in weight and 2% in head circumference in the children of mothers with higher levels of trauma at 5 and 12 months compared to children of mothers with low trauma levels^9,21^. Although these previous studies did not report sex differences, other studies have established that male and female fetuses show different responses to challenges faced during pregnancy, such as maternal anxiety and increased cortisol^22,23^. The scientific literature has identified specific sex-based differences starting from embryonic development, with evidence showing that the placenta in male and female fetuses regulates and expresses genes and proteins differently, thereby directly influencing fetal neurodevelopment^24–26^. Male offspring of anxious pregnant women have higher fetal and birth weights than female offspring as compared to those born to non-anxious mothers, and the same trend was also observed in the first month of life^22,23^. Evidence has also suggested that pregnant women who have experienced ACEs are more likely to develop anxiety during pregnancy, while their offspring are more vulnerable to the effects of the intergenerational transmission of the trauma^27^. However, no study has yet investigated the effects of maternal ACEs on the weight gain of the offspring in the first months of life according to infant sex.

Studies of samples in Brazil, a low-middle-income country (LMIC), showed that > 80% of inhabitants experienced one or more ACEs throughout their lives, which is higher than the rates reported in developed countries (under 50%)^28–31^. According to the Brazilian Institute of Geography and Statistics^32^, the Brazilian population predominantly comprises individuals of black or multiracial ethnic backgrounds. In the Brazilian context, socioeconomic and racial profiles are important factors influencing the occurrence of ACEs, with one study showing that black and multiracial children aged < 18 years were more likely to have ≥ 4 ACEs compared with white individuals^28^.

Recent reports (2020–2023) by Brazilian public security agencies on violence against children and adolescents (0–17 years) revealed an increase in the reporting of the incidence of these practices^33,34^. Further, reports of child neglect and child maltreatment increased by 14% and 13%, respectively^34^. Nevertheless, studies investigating correlations between maternal ACEs and gestational outcomes remain scarce, particularly in LMIC, including Brazil, with admixed populations and different vulnerabilities. Thus, our sample has different racial and socioeconomic features than previous studies evaluating ACEs, which predominantly involved high-income and predominantly White populations^35^. Therefore, this study aimed to evaluate whether maternal ACEs are associated with offspring weight gain in the first 2 months of life, and if such an association depends on the offspring’s sex.

## Methods

### Sample

The maternal inclusion criteria were as follows: (1) living at high-risk and low-resource settings in the cities of Guarulhos and São Paulo (Brazil); (2) users of the Brazilian public healthcare system (*Sistema Único de Saúde*, SUS); (3) age 18–38 years old; (4) between 25th–39th gestational weeks; (5) able to read and understand the informed consent form and to provide written informed consent for partaking in the study.

The maternal exclusion criteria were: (1) high-risk pregnancy; (2) BMI (Body Mass Index) > 30; (3) severe psychiatric conditions (e.g. schizophrenia, persistent delusional disorder, bipolar disorder, obsessive–compulsive disorder, dementia, and suicidal ideation); (4) history of head trauma with brain injury, treatment for epilepsy, and/or neurosurgery; (5) decompensated clinical diseases requiring intensive treatment; (6) illicit drug use (except cannabis); (7) presence of toxoplasmosis, other, rubella, cytomegalovirus, and herpes (also known as TORCH) infections.

Offspring were enrolled at birth after their mothers confirmed their interest in continuing the study. The exclusion criteria for offspring were: (1) prematurity (born before the 37th gestational week), (2) low-weight birth (> 2.5 kg), (3) 5-min APGAR score < 7, (4) admission to the neonatal intensive care unit, (5) presence of kernicterus or inborn errors of metabolism.

A total of 352 mother–child dyads comprising pregnant women with low-risk pregnancies, both with and without ACEs, and their offspring were enrolled. This research protocol was approved by the Institutional Review Board (IRB) of Duke University (Pro00110664) and Columbia University/New York State Psychiatric Institute (7927). In Brazil, it was sanctioned by the National Research Ethics Committee (CONEP; 78018417.2.0000.5505) and the Research Ethics Committee of Universidade Federal de São Paulo (CEP; 1200/2017). All procedures were conducted in compliance with applicable guidelines and regulations.

In accordance with the principles outlined in the Helsinki Declaration, all maternal participants were provided with comprehensive information regarding the study’s objectives and methodologies, and provided written informed consent for themselves and their newborns to participate. Participants were explicitly informed of their right to withdraw from the study at any stage, with no legal or financial repercussions resulting from their decision to discontinue participation.

### Materials and measures

#### Maternal ACEs, race, maternal educational level, and socioeconomic status

Maternal ACEs were evaluated using the CDC-Kaiser ACE Study Questionnaire^2,36^, administered at the enrollment. Participants were asked to self-report their own race and their infant’s race, educational and socioeconomic status, assessed using the Brazilian Economic Classification Criteria 2019^37^.

#### Anthropometric measurements

The following anthropometric measurements of offspring were taken thrice, after birth, immediately after maternity discharge, and during the first 2–8 weeks of life:

Two pediatric neonatologists with more than 10 years of experience in the field performed the following measurements during the first 2 to 8 weeks of the offspring’s life: (1) body length measurement, using an AVANUTRI portable infantometer, (2) body weight measurement, with the infant undressed, using a HOMEIMAGE baby scale model HI-EB522, and (3) head circumference measurement, using a SECA measuring tape, with the glabella and external occipital protuberance as reference points. The intraclass correlations (ICCs) for two-way mixed measures with absolute agreement were calculated. The reliability between the pediatricians’ measurements was high, with an ICC of 0.99 (n = 25; 95% CI 0.99–1.00) for both measurements.

Information regarding the offspring’s birth and maternity discharge, gestational age, birth weight, length, head circumference, and APGAR score, was obtained from the hospital health records, which had been carefully documented by a medical professional. Data on feeding type (breastfeeding, formula feeding, or mixed feeding) was collected through maternal interviews during the pediatric assessment performed between 2 and 8 weeks of life.

The following formula was used to calculate offspring weight gain: W2-W1/number of days, where W1 is the initial weight of the time interval (maternity discharge day), W2 is the final weight of the time interval (2 to 8 weeks of life), and the number of days corresponds to the time between W2 and W1^38^.

### Statistical methods

To test the association between maternal ACEs (focal predictor) and offspring weight gain (outcome), as well as the potential interaction effect between offspring sex and maternal ACEs on offspring weight gain, we applied unconditional and conditional models (i.e. a moderation model). This moderation model was used to evaluate the dependence of the magnitude of the association between maternal ACEs and offspring weight gain on offspring sex. To deal with missing data, as a sensitivity analysis, we assumed missing at random mechanisms and handled missing data under the full information maximum likelihood (FIML) using Mplus^39,40^, a structural equation modeling software. Therefore, we present estimates considering a complete case analysis (e.g., predictors and outcomes must be complete) and under FIML. All descriptive and frequency analyses were performed using SPSS for Windows (version 21.0; SPSS, Chicago, IL), while the interaction model and plot were generated using PROCESS^41^.

All models were adjusted for maternal education, socioeconomic status, offspring sex, offspring birth weight, day of maternal discharge, age at W2, and feeding type (exclusive breastfeeding as reference category, formula use, and mixed feeding). For statistical modeling, maternal education and socioeconomic are categorical variables. The adopted statistical significance level was set at 0.05, and effect sizes were reported regarding standardized regression coefficients.

## Results

In total, 352 full-term newborns (175 males) and their mothers (mean age 27.24 y/o, SD ± 5.15) enrolled in the Mother Influences on Child Biobehavioral Development Study (Healthy MiNDS) were included in this study. Complete case data, including birth weight, day of maternity discharge, weight at W1, age and weight at W2, weight gain, feeding, maternal ACEs, maternal education, and socioeconomic status, were obtained from 320 participants.

Table 1 presents the final data on infant anthropometrics (weight, length, head circumference), feeding type, offspring sex and race, and birth type. Maternal socio-demographic information (ACEs, race, educational level, and family SES) is presented in Table 2.

**Table 1:** Anthropometric offspring data (weight, length, and head circumference) and feeding type, offspring sex, type of birth, and race.

	N	Minimum	Maximum	Mean	Std. deviation	Count	%
Birth weight (g)	341	2500	4500	3284.21	403.41		
Length at birth (cm)	326	43.00	56.50	48.74	1.84		
Head circumference at birth (cm)	312	29.50	37.50	34.19	1.29		
Weight at hospital discharge day (g)	324	2200	4250	3100.90	391.73		
First 2–8 weeks of life (age at W2)							
Age (day)	328	15.00	54.00	32.06	8.62		
Weight (g)	327	2585	6075	4178.13	644.42		
Length (cm)	328	46.00	59.50	53.07	2.31		
Head circumference (cm)	328	33.60	41.60	37.30	1.45		
Weight gain since hospital discharge (g/day)	320	2.07	76.82	35.63	13.64		
Sex	352						
Female						177	50.30
Male						175	49.70
Race	352						
White						158	44.90
Black						22	6.30
Asian						4	1.10
Mixed-race						168	47.70
Indigenous						0	0.00
Type of birth	328						
Vaginal delivery						202	61.60
Cesarean section						122	37.20
Forceps delivery						4	1.20
Feeding	342						
Breastfeeding						247	72.20
Formula						30	8.80
Mixed (Breastfeeding + Formula)						65	19.00

**Table 2:** Maternal socio-demographic data.

	N	Minimum	Maximum	Mean	Std. deviation	Count	%
Age (years)	352	18	38	27.24	5.15		
ACEs	352	0	10	3.54	2.32		
Race	352						
White						123	34.90
Black						63	17.90
Asian						26	7.40
Multiracial						140	39.80
Indigenous						0	0.00
Education	350						
Elementary school complete						4	1.10
Middle school incomplete						6	1.70
Middle school complete						9	2.60
High school incomplete						64	18.20
High school complete						140	38.90
Higher Education incomplete						53	15.10
Higher Education complete						75	21.40
Socioeconomic class and status	350						
U$ 4745,29 (A1)						2	0.60
U$ 2094,47 (B1)						4	1.10
U$ 1047,62 (B2)						28	8
U$ 572,96 (C1)						72	20.60
U$ 324,70 (C2)						125	35.70
U$ 133,66 (DE)						119	34,00

### Unconditional models

The results of the complete case (N = 320) analysis showed that a higher number of reported maternal ACEs was associated with a greater increase in offspring weight gain (1.19 g/day, unstandardized regression coefficient). Overall, male offspring gained an average of 4.98 g/day more than female offspring. Infants who were mixed-fed had a weight gain 4.95 g/day lower than those who were exclusively breastfed. Estimates derived from the FIML approach (N = 352, presented at the bottom of Table 3), align with those from the complete case analysis, further supporting the model robustness (Table 3).

**Table 3:** Sensitivity analysis for the unconditional regression models comparing complete case analysis and full-information maximum likelihood.

Model	Covariates	Unstandardized coefficients		Standardized coefficients			95,0% confidence interval for B	
		B	Std. error	Beta	t	*p*-value	Lower bound	Upper bound
Complete case analysis (N = 320)	Mother’s ACEs	1.194	0.318	0.205	3.759	< 0.001	0.569	1.819
	Birth weight	− 0.002	0.002	− 0.056	− 1.008	0.314	− 0.006	0.002
	Male	4.983	1.529	0.182	3.259	0.001	1.974	7.991
	Formula (breast feeding ref)	− 1.353	2.669	− 0.028	− 0.507	0.613	− 6.604	3.899
	Mixed (breast feeding ref)	− 4.956	1.899	− 0.143	− 2.609	0.010	− 8.693	− 1.219
	Education	0.584	0.629	0.053	0.928	0.354	− 0.654	1.822
	Socioeconomic status	− 0.334	0.784	− 0.025	− 0.426	0.671	− 1.876	1.209
	Age at W2	0.130	0.087	0.081	1.491	0.137	− 0.042	0.302
	Day of maternal discharge	− 0.691	1.185	− 0.032	− 0.583	0.560	− 3.022	1.641
Full-information Maximum Likelihood (N = 352)	Mother’s ACEs	1.180	0.312	0.203		< 0.001	0.569	1.791
	Birth weight	− 0.002	0.002	− 0.052		0.341	− 0.005	0.002
	Male	4.993	1.497	0.183		0.001	2.059	7.928
	Formula (breast feeding ref)	− 1.376	2.620	− 0.029		0.599	− 6.512	3.759
	Mixed (breast feeding ref)	− 4.846	1.857	− 0.140		0.009	− 8.486	− 1.205
	Education	0.639	0.615	0.059		0.299	− 0.567	1.845
	Socioeconomic status	− 0.205	0.755	− 0.015		0.786	− 1.686	1.275
	Age at W2	0.141	0.085	0.089		0.097	− 0.025	0.307
	Day of maternal discharge	− 0.550	1.152	− 0.026		0.633	− 2.808	1.707

### Conditional models

**Fig. 1:**
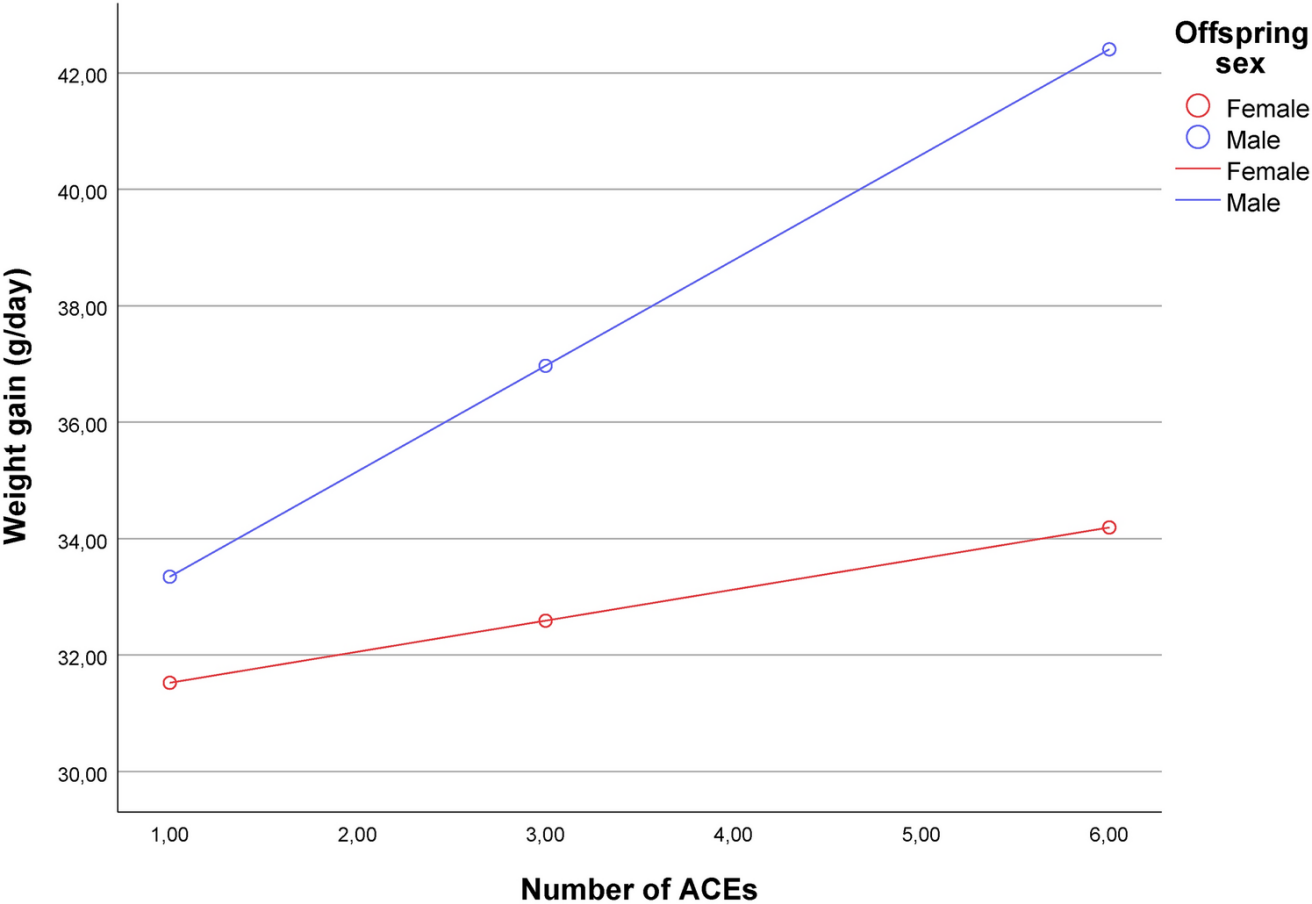
Interaction plot showing the effects of ACEs on weight gain by offspring sex. The three individual points show the interaction plots using conditioning values at the 16th, 50th, and 84th percentiles.

The test for the interaction effect using the complete case analysis approach showed that maternal ACEs and offspring sex could not independently predict offspring weight gain (F_(1,309)_ = 4.45, *p* = 0.0357, R-square change for the interaction = 0.0128), as the association between maternal ACEs and offspring weight gain was positive only among male offspring (unstandardized coefficient_(male)_ = 1.82, SE = 0.438, *p* < 0.001). Consequently, the above-described unconditional model could lead to incorrect inferences regarding the relationships between ACEs and sex on weight gain. Consequently, the nuanced association is best captured and interpreted through a conditional model. In other words, the association between ACEs and gained weight is conditioned to the infant’s sex, where the association between ACEs and weight gain is positive only among males, showing that, depending on the sex, the higher the number of ACEs, the larger is the increasing of weight gain. If the ACEs score increases by 1 point (for example, from 0 to 1), weight gain would increase by 1.8 g/day. Among female offspring, the interaction effect was neither significant nor relevant (unstandardized coefficient_(female)_ = 0.49, SE = 0.457, *p* = 0.285). These results (Fig. 1) showed that the female offspring group had a less stepped line, while the male offspring group showed a positive slope. Interaction effects under FIML were found to converge with complete case analysis (outputs available under request), (Interaction’s regression coefficient = 1.29, *p* = 0.038).

## Discussion

This study showed that the association between maternal ACEs and offspring weight gain during the first two months of life in an admixed population from a LMIC is conditioned to offspring sex with males experiencing greater daily weight gain in the context of maternal ACEs. Additionally, offspring receiving mixed feeding had lower weight gain than those fed via a single method.

The current WHO guidelines indicate that children’s daily weight gain in the first 3 months after birth should range from 25-30 g/day^19^. However, infants in our cohort had a higher average daily weight gain of 35 g/day, which may be a reflection of the increase in weight gain influenced by maternal ACEs, a factor that could contribute to the development of childhood overweight and obesity.

One prior study involving 100 mother-infant dyads reported that the offspring of mothers who experienced childhood trauma were bigger at 5 and 12 months of age compared to those of mothers without trauma ^9^. This difference may be attributed to epigenetic changes and the intergenerational transmission of trauma, potentially aligning with theories concerning the pace-of-life syndrome, which posits that hostile and inhospitable environments characterized by threats to life may prompt a faster developmental trajectory^23,42,43^. It is also important to consider the life history theory, which posits that metabolic resource expenditure can lead to functional trade-offs due to the physiological architecture of the human body, which seeks to maintain homeostasis^44,45^. In this context, this theory predicts that the early-life signals of mortality or environmental unpredictability may influence faster developmental trajectories, serving as a basis for longitudinal studies investigating the effects of childhood adversities on postnatal outcomes^44^.

A cohort study conducted on a rural population in Pakistan examined the impact of maternal ACEs (with an average of 2.3 ACEs in the sample) on offspring development at 36 months of age^46^. This study assessed child growth using WHO z-scores, in addition to fine motor development and receptive language skills, with results indicating that maternal ACEs were associated with better fine motor development and receptive language skills, as well as poorer socio-emotional and behavioral outcomes^46^. Additionally, the study found that as the number of ACEs increased, the effect on child growth became more pronounced, although no statistically significant relationship was observed in the study sample^46^. The present study supports this evidence, showing that the impact of maternal ACEs (with an average of 3.5 ACEs in the sample) could intensify with their magnitude, as reflected in the observed increase in infant weight, particularly among males.

Additionally, one study involving African American mother–offspring dyads evaluated the effects of maternal ACEs on the development of psychopathology in offspring, finding that male offsprings were more prone to displaying externalizing symptoms of psychopathologies related to maternal ACEs^25^. Maternal ACEs can also significantly impact offspring development through changes in the functioning of the hypothalamic–pituitary–adrenal axis and pro-inflammatory cytokines levels^11,23,25^. Indeed, one recent review indicated that the male offspring of mothers with a history of childhood trauma may be more sensitive to dysregulation of the hypothalamic–pituitary–adrenal axis during fetal development^23^. As this axis is responsible for integrating diverse functions between the nervous and endocrine systems, this could compromise development and could explain how maternal trauma and stress could interfere with offspring weight gain, possibly contributing to neurophysiological dysfunctions in adult life^25^. These results are consistent with our findings, showing that male offspring are more sensitive to maternal ACEs, resulting in higher weight gain in early life.

Differences in the regulation and expression of genes, steroids, proteins, and placental structure between males and females are well recognized^24,47^. Male offspring develop strategies to maintain constant growth in the face of an adverse intrauterine environment. They are more susceptible to environmental effects, such as increased levels of maternal cortisol. Conversely, female offspring can adapt to the inhospitable intrauterine environment, via a mechanism involving growth rate/size reduction without developing Intrauterine Growth Restriction (IUGR), resulting in a higher survival rate^24,47,48^. This discrepancy contributes to the higher prematurity and fetal mortality rates in male fetuses, which are also impacted by events that may affect mothers throughout life and pregnancy^48,49^. This potentially explains why maternal ACEs have a greater effect on weight gain in male offspring.

Our data indicate that maternal ACEs are associated with weight gain in male offspring, potentially due to the greater exposure of male fetuses to the effects of maternal ACEs. These results are in line with the life history theory, which posits the existence of a compensatory response to the challenges faced during pregnancy^44^. This compensatory mechanism could manifest itself in an increase in weight gain during the neonatal period in term newborns. Rapid infant weight gain can have a positive effect in premature newborns; however, in full-term newborns it has been linked with a higher risk of health problems, including obesity^50^.

Another study evaluating the effects of maternal ACEs, pre-pregnancy, and gestational body mass index (BMI) on the risk of obesity in preschool-aged offspring found that the offspring of mothers who experienced childhood physical abuse were at a higher risk of obesity at 2–5 years of age, regardless of maternal BMI^10^. A positive correlation has also been found between maternal ACEs, interleukin-6 (a pro-inflammatory cytokine), and obesity in offspring aged 9 years^11^. Moreover, rapid early weight gain in offspring has been strongly associated with cardiovascular and metabolic risks and childhood obesity. Additionally, maternal ACEs may impact offspring weight gain and the aforementioned outcomes through changes in metabolic pathways, inflammatory factors, glucose metabolism, and adipose tissue formation^10,11,51,52^. These findings indicate that the effects of maternal ACEs could be important from the early stages of offspring development. In summary, our existing results emphasize the importance of closely caring for and monitoring the offspring of mothers who experienced ACEs, including in the first months of their lives.

The existing literature on the impact of ACEs on adults has revealed sexual and racial disparities in weight gain, with direct implications on obesity^53^. Notably, the black population is more vulnerable than the white population to these effects. Furthermore, men show an increase in waist circumference and central adiposity compared to women^53^. This evidence aligns with our findings, which showed that maternal ACEs influence offspring weight gain differently by sex, with males being more susceptible. Additionally, one study conducted in the United States examined the impact of maternal ACEs on birth outcomes^54^. This study found that women who identified as Black or African-American exhibited a negative association between the age of menarche and infant cephalization, particularly among those with a high number of ACEs. In contrast, women with a low ACE score did not show any such relationship^54^. This finding further underscores the relevance of our study, which was conducted in Brazil, which encompasses a mixed-race population consisting of approximately 60% women who self-identified as Black or multiracial.

The current consensus is that exclusive breastfeeding in the first 6 months of life is the optimal form of feeding^12,14,52^. Further, postpartum depression may be indicative of early cessation of exclusive breastfeeding, while promoting this practice in the long term could help mitigate depressive symptoms^55,56^. In the present study, 72% of offspring were breastfed exclusively. Further, a previous study examining the impact of maternal ACEs on offspring weight gain at 5 months of age in a sample of 100 mother-baby dyads reported that all offspring were exclusively breastfed in the first few months of life^9^. Our analysis indicated that offspring that received a mixed diet (breast milk with infant formula) showed lower weight gain compared with those who received only breast milk, possibly due to the physicians’ recommendations for mothers to supplement the diets of specific offspring who show weight gain below expectations with infant formula. As the weight gain assessment in our study was performed between the first 2 and 8 weeks of life, this may explain the contrast with other studies, which showed that the use of formula feeding + breastfeeding impacts rapid weight gain from the age of 3 months^15^.

This study had several limitations. First, we relied on retrospective reporting of ACEs about adversities that occurred during mothers’ childhood. Further, this study only included full-term newborns weighing > 2,500 g, excluding premature and low birth weight. This was performed to isolate the direct effects of maternal ACEs on offspring development; however, it may have prevented the assessment of potential associations between low birth weight and maternal ACEs. Furthermore, employing a variable encompassing various traumas precluded us from determining the specific type of trauma most strongly associated with neurobiological alterations in weight gain. Weight gain appears to correlate cumulatively with the number of maternal ACEs. Additionally, our model did not account for pre- and post-gestational maternal nutrition, gestational weight gain (GWG), maternal or infant cortisol levels, or inflammatory markers such as cytokines, all of which may be directly involved in weight gain.

Nevertheless, this study has several notable strengths. Firstly, our cohort aimed to better understand the effects of maternal ACEs on offspring neurodevelopment by including only low-risk pregnant women with a BMI under 30, thus excluding maternal obesity as a potential confounder. We further controlled for birth weight and infant age, as well as the type of feeding provided during the assessment period. Our data support the hypothesis that maternal ACEs independently influence postnatal weight gain in offspring during the first months of life, with significant differences observed based on sex. Furthermore, the target population (primarily Black and mixed-race individuals) differed substantially from the predominantly White populations enrolled in previous studies, adding diversity and heterogeneity to the current research. As such, this study enhances the literature on maternal ACEs and their potential impact on offspring.

## Conclusion

Overall, the findings of the present study suggest that maternal ACEs are associated with offspring weight gain, with variations based on the infant’s sex, in an admixed Brazilian cohort. Specifically, a higher number of maternal ACEs correlated with increased weight gain during the first two months of life, particularly in male infants. This early weight gain may signal potential future metabolic health issues, including the risk of overweight and childhood obesity, thus underscoring the need for increased attention from the pediatric clinical community. In conclusion, our findings support the intergenerational transmission of maternal trauma in a mixed-race Brazilian cohort, and suggest that future research should explore the underlying epigenetic and metabolic mechanisms driving the observed increase in weight gain.

## Data Availability

The raw data supporting the findings of this study are available from the corresponding author (AJ) upon request. Raw data are not publicly available due to restrictions in the written consent signed by the participants of our study.

## Abbreviation

ACEs: Adverse childhood experiences
